# Down‐regulating Myoferlin inhibits the vasculogenic mimicry of melanoma *via* decreasing MMP‐2 and inducing mesenchymal‐to‐epithelial transition

**DOI:** 10.1111/jcmm.13455

**Published:** 2017-11-22

**Authors:** Wenxue Zhang, Ping Zhou, Ai Meng, Rongxin Zhang, Yan Zhou

**Affiliations:** ^1^ Tianjin Medical University General Hospital Tianjin Medical University Cancer Institute and Hospital School of Basic Medical Sciences School and Hospital of Stomatology Tianjin Medical University Tianjin China

**Keywords:** myoferlin, vasculogenic mimicry, MMP‐2, MET, melanoma

## Abstract

Vasculogenic mimicry (VM) constitutes a novel approach for tumour blood supply and contributes to tumour metastasis and poor prognosis in patients with melanoma. Myoferlin (MYOF), a type II membrane protein involved in membrane regeneration and repair, is elevated in several malignant tumours, especially in advanced melanomas. This study aims to investigate the role and mechanism of MYOF in the regulation of VM. VM structures were found in 14 of 52 tested melanoma samples, and high MYOF expression correlated with VM structures. According to Kaplan–Meier survival curves, VM channels and elevated MYOF expression both correlated with poor prognosis in melanoma patients. Down‐regulation of MYOF by siRNA severely impaired the capability of A375 cells to form VM structures *in vitro*. Further studies demonstrated MYOF knockdown inhibited cell migration and invasion, which is required for VM formation, *via* decreasing MMP‐2 expression as evidenced by Western blotting, RT‐RCP and ELISA results. SB‐3CT, a specific inhibitor of MMP‐2, showed similar inhibiting effects with siMYOF, further supporting that MYOF down‐regulation inhibits MMP‐2 expression to affect VM formation. Moreover, MYOF knockdown suppress VM formation by A375 cells by inducing mesenchymal‐to‐epithelial transition (MET). After down‐regulating MYOF, focal adhesions were enlarged and A375 cells developed into a clear epithelial morphology. Such cells acquired the expression of E‐cadherin at adherens junctions along with a loss of mesenchymal markers, such as Vimentin and Twist1. In conclusion, MYOF plays an important role in VM and knockdown of MYOF suppresses VM formation *via* decreasing MMP‐2 and inducing MET in A375 melanoma cells.

## Introduction

Melanoma is the most aggressive type of skin cancer and is always associated with poor prognosis. VM was firstly reported in highly aggressive uveal melanomas by Maniotis in 1999 1. Since then, VM has been found in several types of malignant tumours such as breast cancer, liver cancer, glioma, ovarian cancer, prostate cancer and bidirectional differentiated malignant tumours 2. VM describes the ability of highly invasive melanoma cells to express endothelium‐associated genes and form extracellular matrix (ECM)‐rich vasculogenic‐like channels in three‐dimensional culture 3. VM, formed by tumour cells instead of endothelial cells, invariably indicates poor prognosis and survival in the clinic 4. The molecular mechanisms underlying this unique process are not fully clear, but matrix metalloproteinases (MMPs) and epithelial‐mesenchymal transition (EMT) have been confirmed to play a critical role in the formation of the VM network. MMP‐2 is the most widely recognized modulating molecule involved in VM formation. MMP‐2 colocalized with developing vasculogenic‐like networks and antisense oligonucleotides to the Ln‐5 γ2 chain, and antibody to MMP‐2 inhibited the formation of these networks. Microarray gene chip analyses revealed significant increase in the expression of MMP‐2 in aggressive compared with poorly aggressive melanoma cells, and increased expression of MMP‐2 is required for VM by aggressive melanoma cells 5, 6. The EMT is a process by which epithelial tumour cells gain migratory and invasive properties to become mesenchymal stem cells. EMT regulators and EMT‐related transcription factors are highly up‐regulated in VM‐forming tumour cells. The up‐regulation of EMT‐associated adhesion molecules and other factors can make a contribution in VM‐forming process, which demonstrated that EMT may play a crucial role in VM formation 7, 8.

MYOF, a type II membrane protein, has been confirmed to be involved in membrane regeneration and repair. Recent studies have shown that the expression of MYOF is up‐regulated in several types of cancers, such as breast cancer, lung cancer, pancreatic adenocarcinoma and oropharyngeal squamous cell carcinoma, and high expression of MYOF is associated with poor outcome in those patients 9. The iTRAQ‐based quantitative proteomics reveals MYOF is a novel prognostic predictor in pancreatic adenocarcinoma 10. However, the expression and role of MYOF in melanoma are largely unknown. The elevated MYOF contributes to migration and invasion of cancer cells and promotes tumour metastasis and angiogenesis. MYOF depletion redirects breast cancer cell motility towards collective migration 11 and promotes mesenchymal‐to‐epithelial shape change 12, 13. Moreover, the expression of MYOF is closely associated with the expression of MMPs, such as MMP‐1, MMP‐2 and MMP‐12 12. MYOF regulates cellular lipid metabolism in triple‐negative breast cancer 14 and MYOF depletion suppresses breast cancer cells invasion *via* decreasing MMPs 15. In addition, MYOF plays a key role in VEGFA secretion in human pancreas cancer 16. MYOF expression correlates with VEGFR‐2 expression 17 and MYOF regulates VEGFR‐2 stability and function in non‐small‐cell lung cancer 18. VEGFA and VEGFR‐2 are also critical modulating molecules in VM formation. All above results suggest MYOF may play a role in VM formation in melanoma. Therefore, this study aims to investigate the correlation between MYOF and VM in human melanoma tissues and reveal the underlying mechanisms.

## Materials and methods

### Cells and cell culture

The human cutaneous melanoma cell line A375 was purchased from the Cell Culture Center of Chinese Academy of Medical Sciences (Beijing, China) and cultured according to the instructions. The A375 cell line was characterized by Genetic Testing Biotechnology Corporation (Suzhou, China) using short tandem repeat markers. The cells were cultured in DMEM medium (Gibco, Thermo Fisher Scientific, Waltham, MA, USA) containing 10% foetal bovine serum (FBS) and penicillin/streptomycin (100 U/ml/100 μg/ml) at 37°C in 5% CO_2_.

### Main reagents and antibodies

The following primary antibodies were used: antibodies against MYOF (sc‐376879), Vinculin (sc‐73614) and MMP‐2 (sc‐53630) from Santa Cruz Biotechnology (Dallas, TX, USA); antibodies against Vimentin (ab92547), Twist1 (ab50581) and CD34 (ab81289) from Abcam (Cambridge, MA, USA); antibodies against phospho‐FAK (Y397) (AF3398) and β‐actin (T0022) from Affinity Biosciences (Shanghai, China); and antibody against E‐cadherin (#14472) from Cell Signaling Technology (Danvers, MA, USA). MMP‐2 inhibitor, SB‐3CT (S7430), was obtained from Selleck (Houston, TX, USA). 3‐(4,5‐dimethyl‐2‐thiazolyl)‐2,5‐diphenyl‐2 ‐H‐tetrazolium bromide (MTT) was purchased from Sigma‐Aldrich (St. Louis, MO, USA).

### Immunohistochemical (IHC) staining and assessment

Fifty‐two paraffin‐embedded melanoma tissue specimens and their clinical pathological data were obtained from the Tianjin Huanhu Hospital between 2006 and 2014, and the study was approved by the Institutional Research Committee. Each tissue specimen was reviewed by a pathologist to confirm tumour and determine clinical stage. The experimental procedures and scoring of the IHC assay were performed as described in previous report 19. The following antibodies and dilutions were employed: MYOF (1:100), E‐cadherin (1:100), MMP‐2 (1:200) and CD34 (1:50). PBS was used to replace the primary antibodies for all negative controls. Periodic acid–Schiff (PAS) staining was performed after CD34 staining. PAS‐positive channels exclusively lined by tumour cells without CD34‐stained endothelial cells indicated VM, where red blood cells were present.

### siRNA transfection

MYOF siRNA (siMYOF) (sc‐72293; Santa Cruz) was used to knock down MYOF expression in A375 cells, containing three target‐specific 19–25 nt siRNAs and a scrambled (scr) sequence that will not lead to the specific degradation of any known cellular mRNA. Transfection was performed with the siRNA Reagent System (sc‐45064; Santa Cruz) according to the manufacturer's instructions. At 48 hrs after transfection, the treated cells were harvested for further experiments. The transfection efficiency was determined by Western blotting.

### MTT assay

MTT assay was conducted to evaluate the effect of MYOF on A375 cells proliferation. MYOF‐silenced and scr cells were seeded in 96‐well plates at 2000 cells/well and incubated at 37°C in 5% CO_2_. Subsequently, 20 μl of MTT reagent (10 mg/ml; Sigma‐Aldrich) was added to each well for further 4 hrs incubation. The medium was then discarded, and 150 μl of dimethylsulfoxide (DMSO) was added to each well. The plate was then gently shaken until the purple crystals dissolved. Subsequently, the absorbance of each well was measured at 490 nm using a microplate reader (BioTek Epoch, Winooski, VT, USA).

### Three‐dimensional (3D) cultures

For this assay, 48‐well plates were coated with 120 μl of Matrigel matrix (BD Biosciences, Sparks, MD, USA) diluted with pre‐cooling serum‐free DMEM at ratio of 1:1, pre‐treated on ice for 10 min. and incubated for 1 hr at 37°C. A suspension of A375 cells in 200 μl serum‐free DMEM containing 2 × 10^5^ cells was seeded onto the matrix and incubated at 37°C for 9 hrs. Subsequently, photomicrographs of each well were taken by a computer‐based phase‐contrast microscope (Olympus, Tokyo, Japan). The closed channels in six random fields of each group were counted to quantify VM formation by A375 cells.

### Wound healing assay

Wound healing assays were performed according to the protocol described previously. 20 Briefly, A375 cells pre‐treated with scr or siMYOF were cultured in 60 mm dishes at a density of 8 × 10^5^ cells/well and incubated for 12 hrs to grow a monolayer. Then, a linear scratch wound was created across the middle of each well using a 200‐μl pipette tip. The cells were then incubated in serum‐free medium at 37°C in 5% CO_2_, and the wounds were photographed and measured with an inverted microscope (Olympus). The migration distance was monitored at intervals until the wounds were occluded.

### Transwell assay

Cell invasion was evaluated by Transwell assays. First, 100 μl of serum‐free cell suspension containing 5 × 10^4^ cells was seeded into the upper chamber, which had been coated with 50 μl of Matrigel matrix. Subsequently, 600 μl of DMEM containing 10% FBS was added to the lower chamber. After migrating for 24 hrs incubated at 37°C in 5% CO_2_, the cells were fixed with cold methanol, stained with the Three‐Step Stain Set (370164; Richard‐Allan Scientific, Kalamazoo, MI, USA) and counted using a computer‐based microscopy imaging system. Each experiment was performed three times.

### Adhesion assay

Adhesion assays were performed as described previously 20. Briefly, A375 cells were pre‐treated with scr or siMYOF, washed three times with binding medium (DMEM, 0.1% BSA, and 25 mM HEPES) and then suspended at a density of 3 × 10^5^ cells/ml in serum‐free DMEM. Subsequently, 1 ml of the cell suspension was promptly placed in a 35 mm dish containing a dried glass coverslip coated with 10 ng/ml fibronectin at 4°C overnight. After further incubations for 5, 15 and 30 min., the cells were gently washed, fixed and counted at 400× magnification in five separate fields under a light microscope.

### Immunofluorescent (IF) staining

The morphology of focal adhesions (FA) and E‐cadherin/Vimentin in EMT was stained by immunofluorescence and photographed under a fluorescent microscopy (BX51; Olympus) and a laser scanning confocal microscope (LSCM) (FV1000; Olympus), respectively. Briefly, A375 cells transfected with siMYOF or scr were plated in 12‐well plates containing sterile coverslips, allowed for 24‐hrs growth at 37°C in 5% CO_2_ and starved in serum‐free DMEM for 6 hrs. Subsequently, cells were fixed with 4% paraformaldehyde, quenched with 50 mM NH_4_Cl, permeabilized in 0.2% Triton X‐100 and blocked in 3% bovine serum albumin. Cells were then stained with antibodies against E‐cadherin (1:50) and Vimentin (1:200) or Vinculin (1:200) and probed with either an Alexa Fluor 488—or an Alexa Fluor 568—conjugated secondary antibody. Then the coverslips were sealed with ProLong™ Gold antifade reagent (P36934; Invitrogen, Thermo Fisher Scientific, Waltham, MA, USA) and observed under a fluorescent microscopy or LSCM.

### Western blotting analysis

Western blotting assay was used for assessment of expressions of MMP‐2, p‐FAK^Y397^, Twist1, E‐cadherin and Vimentin in MYOF down‐regulated A375 cells. The cells were lysed on ice for 30 min., and 15 μg of protein per sample was separated using 10% SDS‐PAGE. The separated proteins were then transferred onto PVDF membranes (Millipore, St. Louis, MO, USA). Subsequently, the membranes were blocked with 5% non‐fat milk for 1 hr at room temperature and incubated with diluted primary antibodies overnight at 4°C. The antibodies and dilution factors were as follows: MYOF (1:1000), MMP‐2 (1:1000), p‐FAK^Y397^ (1:3000), E‐cadherin (1:1000), Vimentin (1:1000), Twist1 (1:1000) and β‐actin (1:3000). Secondary antibodies conjugated with HRP were incubated for 1 hr at room temperature the following day. A G‐BOX (Gene Company Ltd, Beijing, China) was used to photograph and analyse bands using ImageJ software.

### Real‐time PCR (RT‐PCR)

Total RNA from A375 cells pre‐treated with siMYOF or scr was extracted by Trizol. Then, cDNA was synthesized using a FastQuant RT kit (TIANGEN, Beijing, China). The following real‐time PCR primers referring to previous report 21 were used: MMP‐2 (Sense: 5′‐GTTTCCATTCCGCTTCCAGG‐3′, Antisense: 5′‐TGCCCTTGATGTCATCCTGG‐3′); GAPDH (Sense: 5′‐GACCTGACCTGCCGTCTA‐3′, Antisense: 5′‐AGGAGTGGGTGTCGCTGT‐3′). All samples were subjected to a 7500 Fast Real‐Time PCR System (Applied Biosystems, Foster, CA, USA) according to the manufacturer's instructions of the real‐time PCR Master Mix (SYBR Green).

### ELISA assay

A375 cells pre‐treated with siMYOF or scr were plated in 60 mm dishes at a density of 6 × 10^5^ cells per dish and cultured in 3 ml DMEM supplemented with 10% FBS for 4 days at 37°C. An aliquot of the cell culture supernatant was removed and assayed for levels of natural total MMP‐2 with the Total MMP‐2 Quantikine ELISA Kit (MMP200; R&D Systems, Inc., Minneapolis, MN, USA) according to its protocol in the manufacturer's instructions. Then, cells were lysed and total protein concentrations were determined by the Pierce™ BCA Protein Assay Kit (#23225; Thermo Fisher Scientific). The content of total MMP‐2 was normalized against total protein to correct the deviation induced by cell amount.

### MMP‐2 specific inhibitor, SB‐3CT

SB‐3CT, a specific inhibitor of MMP‐2, was dissolved in DMSO and used to treat cells for 24 hrs at concentrations of 10, 50 and 250 nM. The cells were then used for 3D cultures, Wound healing assay and Transwell assay. Equivalent DMSO was used as controls. The activity of MMP‐2 and MMP‐9 was assessed by Zymography assays.

Serum‐free conditioned medium was collected for SDS‐PAGE using a 10% polyacrylamide gel containing 0.01% w/v gelatine according to previous reports 7.

### Statistical analysis

All data were analysed with SPSS version 17.0 (SPSS, Chicago, IL, USA). The measured data are expressed as the mean ± S.D. or S.E.M. A *P* value of <0.05 was defined as significant. Differences between two groups were assessed using Student's *t*‐test, whereas multiple groups were compared by anova. Kaplan–Meier survival curve analysis was also performed. Differences in survival curves were evaluated using the log‐rank test.

## Results

### MYOF is overexpressed in melanoma and correlates with VM formation

**Figure 1:**
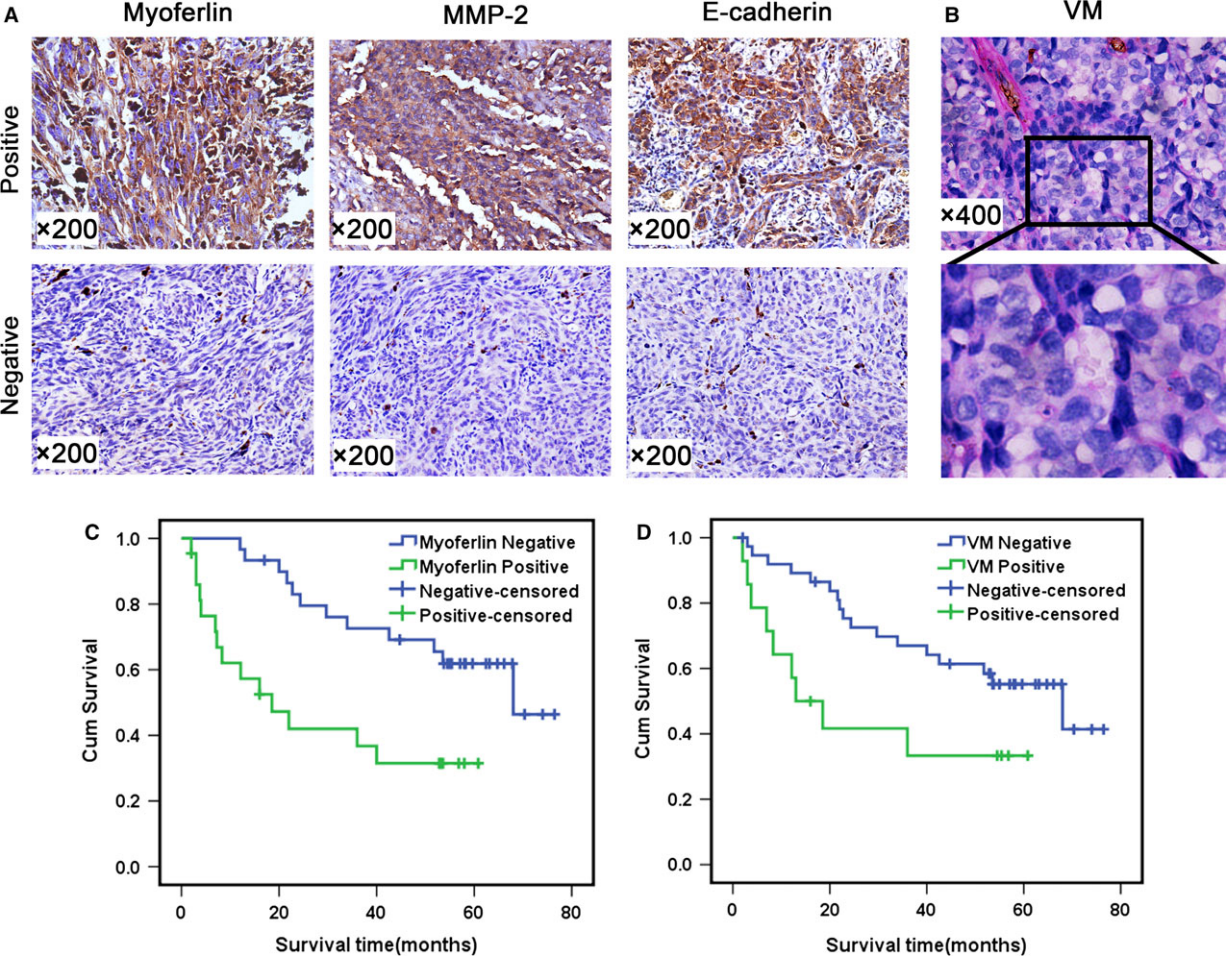
Myoferlin is overexpressed in invasive melanoma and correlated with VM. **A**, Immunohistochemical staining of Myoferlin/MMP‐2/E‐cadherin (200×). **B**, The phenomena of VM (400×) in melanoma. **C**, The expression of Myoferlin correlated with prognosis and survival of melanoma patients. **D**, VM correlated with prognosis and survival of melanoma patients.

The expression of MYOF was determined by IHC in 52 melanoma tissue samples. Specifically, MYOF‐positive staining was found in 22 samples, among which 17 cases were accompanied with recurrence or metastasis, suggesting that MYOF is overexpressed during melanoma tumorigenesis (Table 1, Fig. 1A). VM structures with PAS‐positive and CD34‐negative staining were observed in 11 samples with MYOF overexpression but only three samples with MYOF low expression (*P = *0.001; Table 1, Fig. 1B), indicating that the expression of MYOF closely correlates with VM. Kaplan–Meier survival analysis revealed that patients with elevated expression of MYOF and VM formation have similar poor prognosis and survival (Fig. 1C). Moreover, MMP‐2‐positive staining was found in 19 of 22 samples with MYOF overexpression (*P = *0.022; Table 1, Fig. 1A) and 13 of 14 samples with VM formation (*P = *0.025; Table 2, Fig. 1A), respectively. The staining of E‐cadherin, which is an epithelial marker and negatively correlates with MYOF and VM, was found positive in only one case of the samples with MYOF overexpression (*P = *0.037; Table 1, Fig. 1A), and none of the samples with VM showed E‐cadherin‐positive staining (*P = *0.045; Table 2, Fig. 1A). Thus, MMP‐2 and E‐cadherin were confirmed to be involved in the connection between MYOF expression and VM formation. Taken together, the above results indicate that the expression of MYOF is elevated in melanoma and closely correlates with VM formation. MMP‐2 and E‐cadherin may serve as downstream effectors in the regulation of VM by MYOF.

**Table 1:** The correlation of Myoferlin with the clinicopathological parameter of melanoma, VM and the expression of MMP‐2/E‐cadherin

Variables	Cases	Myoferlin expression		χ^2^	*P* value
		Positive(22)	Negative(30)		
Gender					
Male	36	14	22	0.560	0.454
Female	16	8	8		
Age(years)					
<55	15	7	8	0.164	0.685
≥55	37	15	22		
Tumour size (cm^3^)					
<7.8	31	10	21	3.176	0.075
≥7.8	21	12	9		
Pathological grade					
I–II	22	7	15	1.719	0.190
III–IV	30	15	15		
Recurrence or Metastasis					
Yes	32	17	15	3.989	0.046
No	20	5	15		
VM					
Yes	14	11	3	10.322	0.001
No	38	11	27		
MMP‐2					
Positive	36	19	17	5.255	0.022
Negative	16	3	13		
E‐cadherin					
Positive	9	1	8	4.340	0.037
Negative	43	21	22		

**Table 2:** The correlation of VM with the clinicopathological parameter of melanoma and the expression of MMP‐2/E‐cadherin

Variables	Cases	VM		χ^2^	*P* value
		Yes(14)	No(38)		
Gender					
Male	36	10	26	0.043	0.835
Female	16	4	12		
Age(years)					
<55	15	6	9	1.832	0.176
≥55	37	8	29		
Tumour size (cm^3^)					
<7.8	31	8	23	0049	0.825
≥7.8	21	6	15		
Pathological grade					
I–II	22	4	18	1.481	0.224
III–IV	30	10	20		
Recurrence or Metastasis					
Yes	32	12	20	4.731	0.030
No	20	2	18		
Myoferlin					
Positive	22	11	11	10.322	0.001
Negative	30	3	27		
MMP‐2					
Positive	36	13	23	5.020	0.025
Negative	16	1	15		
E‐cadherin					
Positive	9	0	9	4.010	0.045
Negative	43	14	29		

### Down‐regulation of MYOF inhibits VM formation by A375 cells *in vitro*

**Figure 2:**
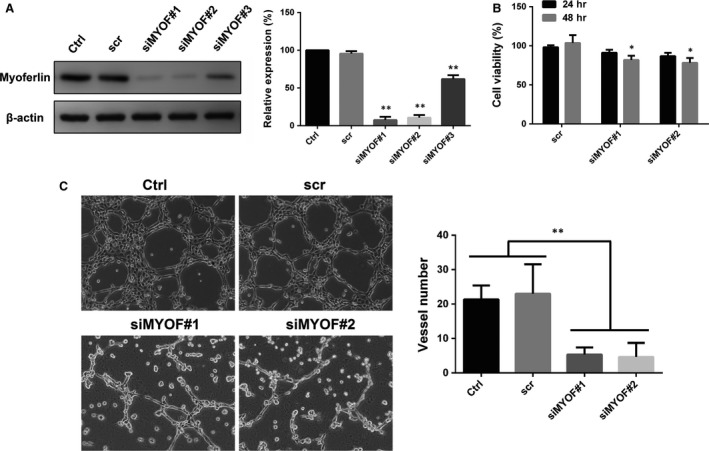
Down‐regulation of Myoferlin disrupted VM structures formation *in vitro*. **A**, Myoferlin was knockdown by siRNA with three different sequences. **B**, Cell proliferation was slightly affected after knockdown of Myoferlin. **C**, Down‐regulating Myoferlin inhibited VM structures formation on Matrigel by A375 cells (100×). Each condition was carried out with three individual replicates. *P < 0.05; **P < 0.01.

To test this hypothesis that MYOF plays an important role in the VM of melanoma cells, a MYOF siRNA kit containing three independent target‐specific siRNAs (#1, #2 and #3) and a scr sequence was purchased from Santa Cruz Inc. and transfected to silence MYOF in A375 cells. Transient transfection with each of the three siRNAs severely decreased MYOF expression in A375 cells (siMYOF#1 and siMYOF#2 both more than 90%; siMYOF#3 about 40%), whereas transfection with the scr sequence did not (Fig. 2A). Two of the siMYOF sequences (#1 and #2) were selected to serve as representatives for later results. To evaluate the effect of MYOF on cell proliferation, MTT assays were performed. As shown in MTT results (Fig. 2B), after knockdown of MYOF, the viability of A375 cells showed no significant difference at 24 hrs and decreased <20% at 48 hrs, respectively, which did not interfere with the subsequent assays of motility properties. This assay demonstrated that the down‐regulation of MYOF slightly affected the proliferation of A375 cells. The ability of A375 cells to form capillary‐like tubes was evaluated *in vitro* by seeding the cells on Matrigel‐coated plates. The down‐regulation of MYOF severely disrupted VM channels formation by A375 cells, as evidenced by most tubes remaining open and an increase in dissociative cells (Fig. 2C). This finding signifies that MYOF plays an important role in VM formation.

### Knockdown of MYOF severely impairs A375 cells migration and invasion *via* decreasing MMP‐2

**Figure 3:**
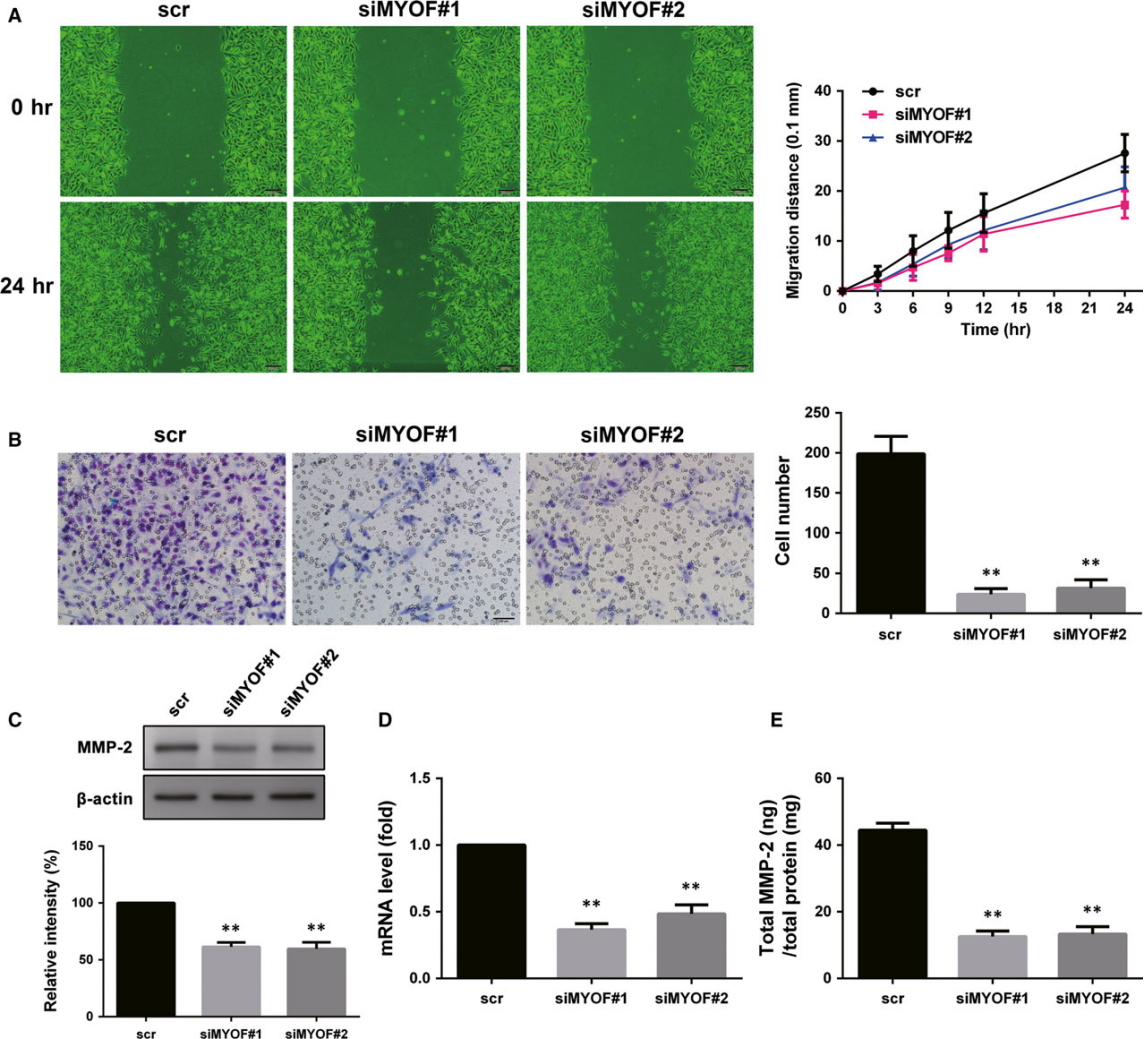
Knockdown of Myoferlin suppressed cell metastasis and MMP‐2 expression. **A**, Migration of A375 cells transfected with siMYOF or scr in Wound healing assay (100×). **B**, Invasion of A375 cells after down‐regulation of Myoferlin was detected by Transwell assay (200×). **C**, The expression of MMP‐2. **D**, mRNA level of MMP‐2 detected by RT‐PCR. **E**, Total MMP‐2 concentrations in cell culture supernates were determined by ELISA. *P < 0.05; **P < 0.01.

Migration and invasion are two critical steps in the motility of tumour cells, and cell motility is required for VM formation. The migration of A375 cells was evaluated *via* the Wound healing assay. As shown in Figure 3A, knockdown of MYOF decreased A375 cells healing rate of the wound, indicating that MYOF indeed affects, albeit slightly, the directional migration of A375 cells. The invasion of A375 cells was then evaluated by the Transwell assay with hanging inserts coated with Matrigel™ matrix. After MYOF knockdown, the number of A375 cells migrating through 8.0 μm PET decreased by about 85% in the invasion assays (Fig. 3B). To further study whether MYOF regulates cell migration and invasion *via* MMP‐2, Western blotting assay, RT‐PCR and ELISA assay were performed, respectively. As shown in Western blotting assay results, the expression of MMP‐2 decreased more than 40% in MYOF down‐regulated cells (Fig. 3C), which correlates with the decreased mRNA levels detected by RT‐PCR (Fig. 3D). ELISA assay was used for the quantitative determination of total MMP‐2 concentrations in cell culture supernates, including active‐, pro‐ and TIMP‐complexed MMP‐2. As shown in Figure 3E, excluding the deviation induced by cell density, the content of MMP‐2 in cell culture supernates decreased by approximately 65% after MYOF knockdown. Taken together, these data show that MYOF knockdown significantly impaired the migration and invasion of A375 cells *via* decreasing the expression of MMP‐2.

### MMP‐2 inhibitor SB‐3CT and siMYOF exhibit similar inhibition of VM formation, migration and invasion of A375 cells

**Figure 4:**
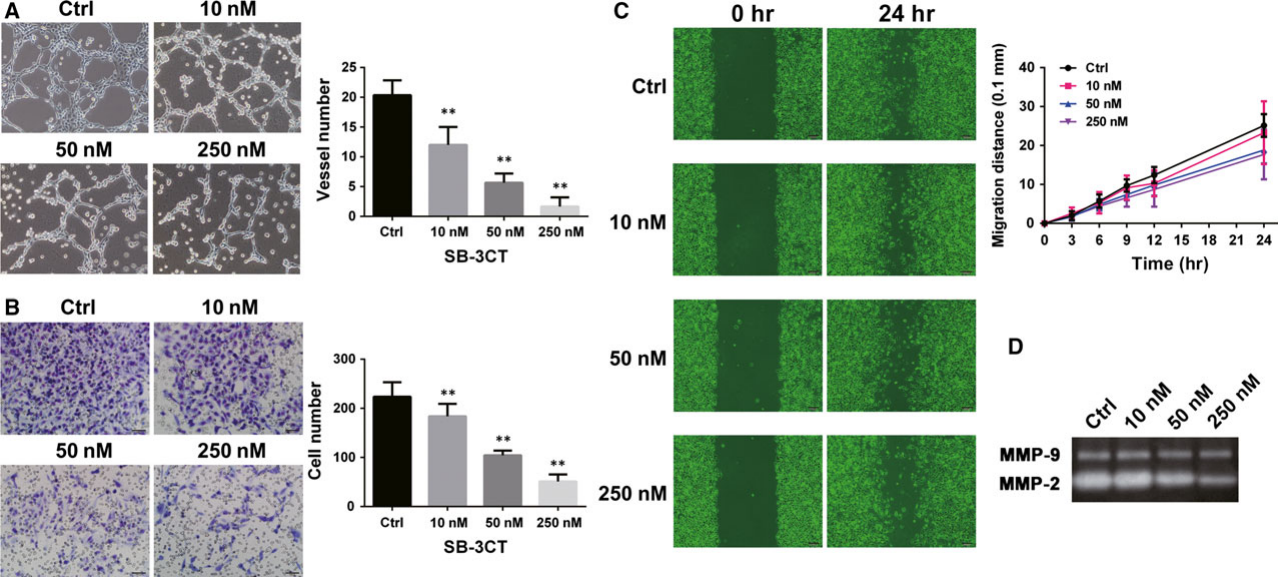
Inhibition induced by siMYOFs is consistent with that by MMP‐2 inhibitor, SB‐3CT. **A**, SB‐3CT inhibited A375 cells forming VM structures *in vitro* (100×). Each condition was carried out with three individual replicates. **B**, Invasion of A375 cells was suppressed by SB‐3CT in Transwell assay (200×). **C**, Migration of A375 cells was affected by SB‐3CT in Wound healing assay (100×). **D**, The activity of MMP‐2 and MMP‐9 secreted by A375 cells treated with different concentrations of SB‐3CT was detected by Zymography assay.*P < 0.05; **P < 0.01.

SB‐3CT is a potent‐specific inhibitor of MMP‐2 and Ki values are 13.9 and 600 nM for MMP‐2 and MMP‐9, respectively 22. To confirm that MYOF regulates VM formation, cell migration and invasion *via* MMP‐2, SB‐3CT was used to treat A375 cells at dose of 10, 50 and 250 nM, which severely inhibited MMP‐2 without significant inhibition of MMP‐9 as confirmed by Zymography assay (Fig. 4D). As shown in Figure 4A, treatment with SB‐3CT significantly disrupted the channels formation by A375 cells in a dose‐dependent manner. After treatment with SB‐3CT, the directional migration of A375 cells in Wound healing assays was slightly inhibited and 250 nM of SB‐3CT decreased the migration distance less than 30% at 24 hrs (Fig. 4C). However, cell invasion, which depends on MMP‐2 in the breakdown of ECM like Matrigel™ matrix, was severely impaired by SB‐3CT in a dose‐dependent manner (Fig. 4B). The results indicate MMP‐2 inhibition can severely disrupt VM formation and impair cell invasion, and the effect is more significant than cell migration, which is consistent with the inhibition effect induced by siMYOF.

### Down‐regulation of MYOF enhances cell adhesion and enlarges FA

**Figure 5:**
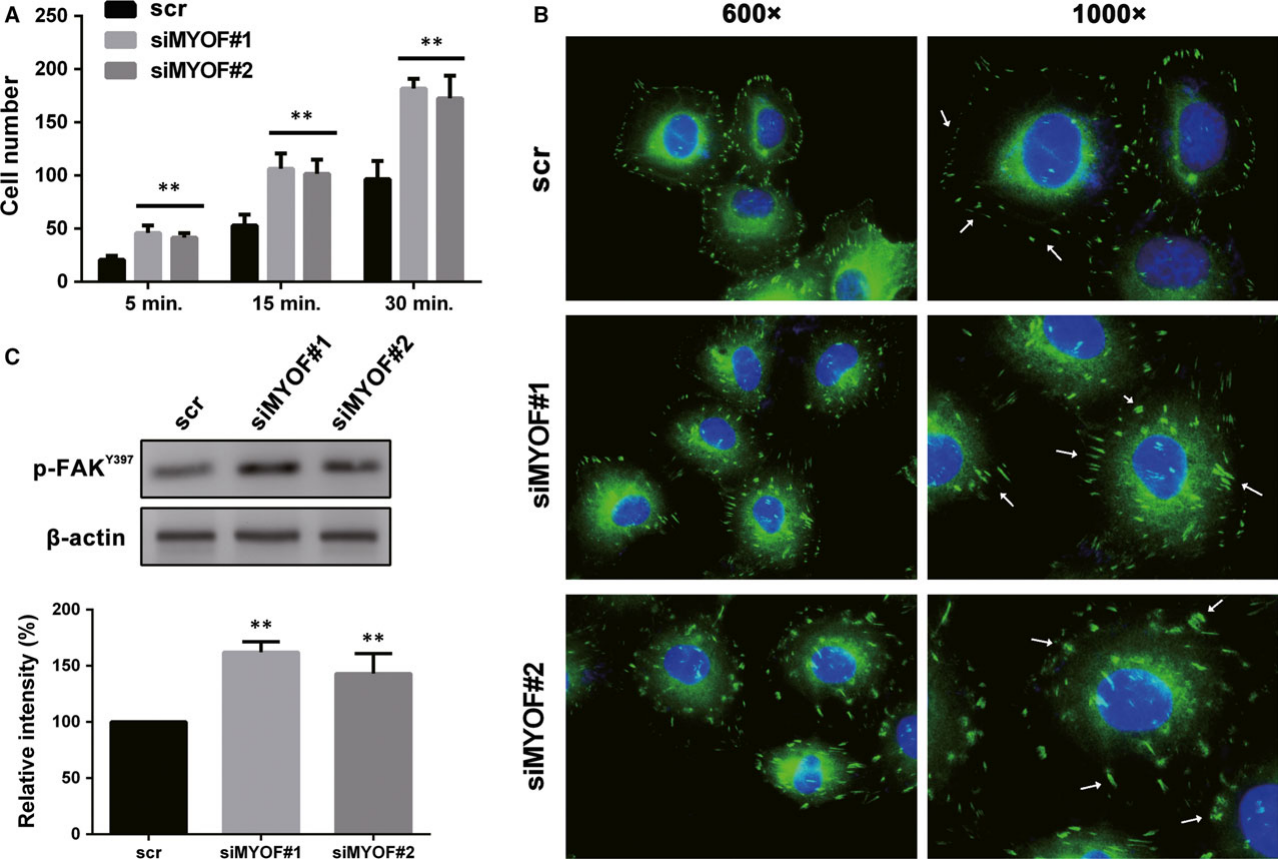
Down‐regulation of Myoferlin enhanced cell adhesion and enlarged FA. **A**, The matrix adhesion of A375 cells was enhanced after down‐regulating Myoferlin. **B**, FA was stained by anti‐Vinculin and captured by fluorescence microscope. (600× and 1000×) **C**, The expression of p‐FAK^Y397^ increased in Myoferlin down‐regulated cells as detected by Western blotting assay. *P < 0.05; **P < 0.01.

The cell dissociation is the first step in EMT. Thus, the enhancement of cell adhesion is important to reflect MET and is analysed by the Cell adhesion assay. As shown in Figure 5A, MYOF down‐regulation significantly increased the number of cells adhering to a fibronectin‐coated coverslip at various time‐points during the adhesion assay. FAs were then stained with immunofluorescence against Vinculin. As arrows indicating in Figure 5B, FAs in MYOF‐knockdown cells were dramatically enlarged in size, compared with those in scr‐transfected cells. To further confirm the effect of MYOF on FAs by biochemical criteria, the levels of p‐FAK^Y397^ were monitored by Western blotting assays, an activation site of FAK that becomes phosphorylated upon FAs assembly and correlates with the number of FAs 23. As Figure 5C shows, the phosphorylation of FAK^Y397^ increased by about 60% and 45% in siMYOF#1 and siMYOF#2 treated cells, respectively. All the results signify that down‐regulation of MYOF promotes cell adhesion and enlarges FAs, suggesting MYOF may play a role in EMT.

### Knockdown of MYOF induces MET in A375 cells

**Figure 6:**
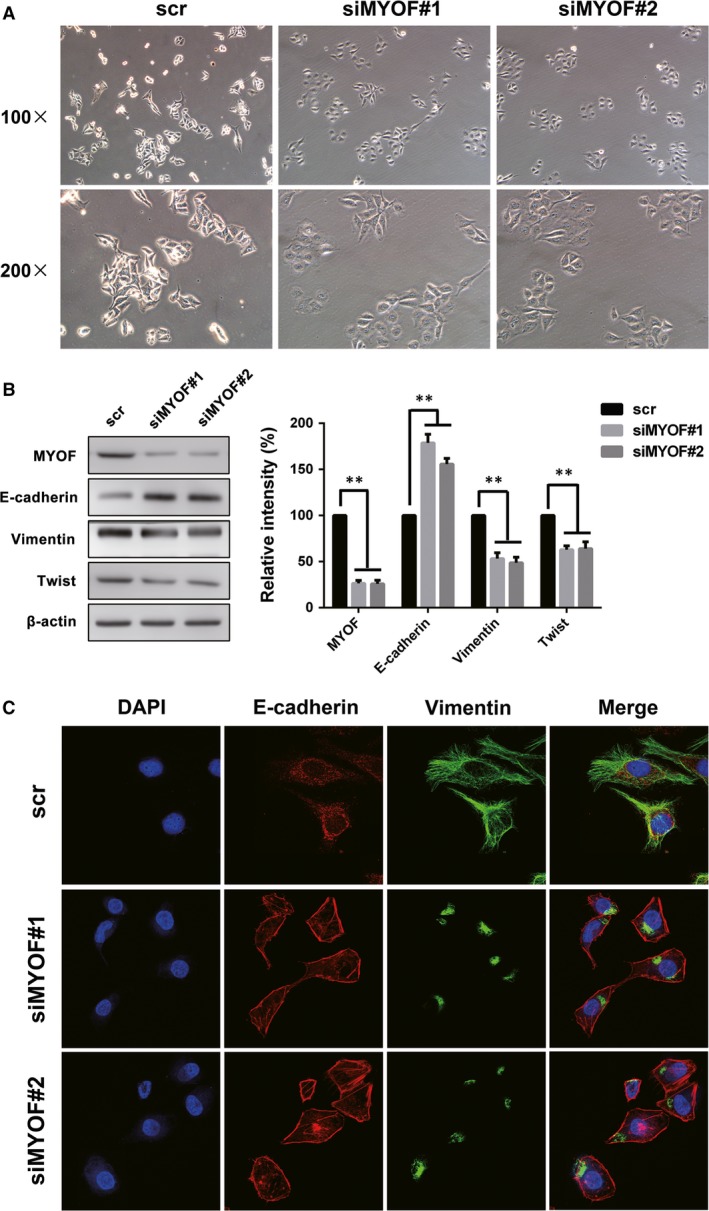
Down‐regulation of Myoferlin induced MET in A375 cells. **A**, The cell morphology transformation induced by siMYOFs. **B**, The expression of E‐cadherin, Vimentin and Twist in Myoferlin down‐regulated cells. **C**, The expression and subcellular localization of E‐cadherin and Vimentin were observed by laser scanning confocal microscope. *P < 0.05; **P < 0.01.

To further test the role of MYOF in EMT regulation, cell morphology was observed under an optical microscope and the expression of EMT markers was monitored by Western blotting assay and IF staining. E‐cadherin plays a key role in cellular adhesion and is an epithelial marker that disappears as a hallmark of EMT 24. Vimentin is a type III intermediate filament protein that is expressed in mesenchymal cells. Twist1 is a prominent EMT regulator involved in the suppression of E‐cadherin expression and the induction of VE‐cadherin up‐regulation 25, 26, 27. A375 cells tend to be in the form of mesenchymal morphology and lack expression of E‐cadherin at prominent cell junctions 28. As shown in Figure 6A, MYOF down‐regulation induced the formation of islands of cells with the characteristic cobblestone morphology of epithelial cells. The expression of E‐cadherin, a key epithelial protein, increased by more than 65% at adherens junctions, while the levels of mesenchymal markers, such as Vimentin and Twist1, decreased by about 50% and 40%, respectively (Fig. 6B and C). These results show that MYOF knockdown induced MET in A375 cells by both biochemical and cell biological criteria.

## Discussion

VM is a marker of poor prognosis and contributes to tumour metastasis in several types of cancers, such as prostate cancer, 29 hepatocellular carcinoma, 30 gastric carcinoma 31 and invasive melanoma. Although the molecular mechanisms underlying the process are not fully clear, MMP‐2 and EMT have been confirmed to play a critical role in VM formation. MYOF, a membrane protein associated with membrane regeneration and repair, has been reported overexpressed in many cancers and promotes tumour metastasis resulting in poor outcome in patients. Further studies reveal that MYOF mainly contributes to metastasis *via* enhancing the expression of MMP‐2 and regulating EMT in breast cancer cells 12. Therefore, the expression of MYOF in melanoma tissues and the connection between MYOF and VM were examined in this study. The pathological investigation showed that the expression of MYOF was dramatically elevated in melanoma tissues. The melanoma tissues overexpressing MYOF are prone to form VM channels, and this formation is accompanied by an increase in MMP‐2 secretion and loss of E‐cadherin at adherens junctions. Three‐dimensional cultures *in vitro* also indicated that down‐regulating MYOF severely impaired the formation of VM tubes by A375 cells, further supporting the hypothesis that MYOF regulates VM formation.

Cell proliferation and motility are two critical factors affecting VM formation by tumour cells. The effect of MYOF knockdown on cell proliferation is quite slight as indicated by MTT assays, probably because MYOF is a type II membrane protein and not involved in pivotal proliferation‐related signal pathways. As reported previously, MYOF depletion in MDA‐MB‐231 cells significantly decreased the secretion of MMPs‐1, 2, 3, 8 and 16 12, among which MMP‐2 is a recognized promotor of VM formation. Our results indicate that MYOF depletion in A375 cells also decreases the expression of MMP‐2 at both protein and mRNA levels. MYOF depletion inhibits MMP‐2 secretion, probably because MYOF depletion results in marked alteration of endosomal system and causes impaired vesicle traffic, on which MMP‐2 secretion is dependent 32. The decrease in MMP‐2 induced by MYOF depletion then affects cell migration and severely suppresses cell invasion, which are required for VM formation by tumour cells. According to previous report, 11 MYOF‐depleted MDA‐MB‐231 cells exhibit reduced average migration velocity and accumulated distance. However, increased directional persistence made up the disparity in Euclidean distance, which was directly observed in Would healing assay. In our results, melanoma cells migration distance observed in Would healing assay was not robustly decreased after MYOF depletion, which correlated to that observed in breast cancer cells. The migration velocity, directionality and accumulated distance were not compared in this study. SB‐3CT is an effective and selective inhibitor of gelatinase, including MMP‐2 and MMP‐9. It either does not inhibit or inhibits poorly other MMPs, such as MMP‐3, MMP‐7 and MMP‐1 33. The Zymography assay results also indicate a severe inhibition of MMP‐2 by SB‐3CT. The inhibition effects on VM structures, cell migration and invasion induced by SB‐3CT are consistent with those induced by siMYOF, which further supports that MYOF regulates VM formation *via* MMP‐2.

EMT is a process by which epithelial cells lose their cell polarity and cell–cell adhesion, and gain migratory and invasive properties to become mesenchymal cells. Previous studies have signified that tumour cells depend on EMT to acquire stem‐like properties and then form VM structures, especially in melanoma. The cell adhesion enhancement is the first step of MET. MYOF depletion induced the increased size of FAs and elevated the phosphorylation of FAK^Y397^, an activation site of FAK correlates with the FAs assembly, which is consistent with previous results in breast cancer cells 34. The results suggest that MYOF depletion enhance cell adhesion and may induce MET in A375 cells. After knockdown of MYOF, A375 cells display an obvious epithelial morphology and such cells acquired the expression of E‐cadherin at adherens junctions along with a loss of mesenchymal markers, such as Vimentin and Twist1. Western blotting results also confirm the elevated expression of E‐cadherin and the reduction in Vimentin and Twist1 expression, which is similar to the effect of MYOF in breast cancer cells 12. All these results signify that MYOF depletion inhibits VM formation *via* inducing MET in A375 cells.

In conclusion, this study is the first to demonstrate that MYOF plays an important role in melanoma VM formation by regulating the expression of MMP‐2 and promoting EMT, indicating that MYOF may be a potential biomarker for VM formation in melanoma and can be used to predict the prognosis of melanoma patients.

## Conflict of interest

The authors confirm that there is no conflict of interests.
